# Comparative Study of Ergonomics in Conventional and Robotic-Assisted Laparoscopic Surgery

**DOI:** 10.3390/s24123840

**Published:** 2024-06-14

**Authors:** Manuel J. Pérez-Salazar, Daniel Caballero, Juan A. Sánchez-Margallo, Francisco M. Sánchez-Margallo

**Affiliations:** 1 Bioengineering and Health Technologies Unit, Jesús Usón Minimally Invasive Surgery Centre, ES-10004 Cáceres, Spain; mjperez@ccmijesususon.com (M.J.P.-S.); dcaballero@ccmijesususon.com (D.C.); 2 Scientific Direction, Jesús Usón Minimally Invasive Surgery Centre, ES-10004 Cáceres, Spain; msanchez@ccmijesususon.com

**Keywords:** minimally invasive surgery, urology, gynecology, general surgery, simulation setting, wearable device, motion analysis, stress level, localized muscle fatigue, muscle activity

## Abstract

BACKGROUND: This study aims to implement a set of wearable technologies to record and analyze the surgeon’s physiological and ergonomic parameters during the performance of conventional and robotic-assisted laparoscopic surgery, comparing the ergonomics and stress levels of surgeons during surgical procedures. METHODS: This study was organized in two different settings: simulator tasks and experimental model surgical procedures. The participating surgeons performed the tasks and surgical procedures in both laparoscopic and robotic-assisted surgery in a randomized fashion. Different wearable technologies were used to record the surgeons’ posture, muscle activity, electrodermal activity and electrocardiography signal during the surgical practice. RESULTS: The simulator study involved six surgeons: three experienced (>100 laparoscopic procedures performed; 36.33 ± 13.65 years old) and three novices (<100 laparoscopic procedures; 29.33 ± 8.39 years old). Three surgeons of different surgical specialties with experience in laparoscopic surgery (>100 laparoscopic procedures performed; 37.00 ± 5.29 years old), but without experience in surgical robotics, participated in the experimental model study. The participating surgeons showed an increased level of stress during the robotic-assisted surgical procedures. Overall, improved surgeon posture was obtained during robotic-assisted surgery, with a reduction in localized muscle fatigue. CONCLUSIONS: A set of wearable technologies was implemented to measure and analyze surgeon physiological and ergonomic parameters. Robotic-assisted procedures showed better ergonomic outcomes for the surgeon compared to conventional laparoscopic surgery. Ergonomic analysis allows us to optimize surgeon performance and improve surgical training.

## 1. Introduction

Laparoscopic surgery is a surgical technique with well-known benefits for the patient compared to traditional open techniques, such as the reduction of tissue trauma, postoperative pain, or hospital stay, among others. However, it is a technique with a steep learning curve, and it is physically and mentally demanding for the surgeon. As a result, the onset of musculoskeletal problems in surgeons is common, mainly due to the ergonomic deficiencies of the equipment and the surgical work environment [1].

The advantages of surgical robotics include tools to improve the surgical precision, a better visual perception of the surgical field thanks to three-dimensional imaging, and a more comfortable posture for the main surgeon at the console, who can operate in a seated position. However, there is still scarce scientific evidence that surgical robotics significantly improves surgeon ergonomics over conventional laparoscopic surgery [2].

On the other hand, the technical difficulty of laparoscopic surgery, whether robotic-assisted or not, together with the complexity of certain surgical procedures, leads to a considerable increase in the surgeon’s stress levels. This can negatively affect the quality of the surgery and the patient’s surgical outcome [3]. Being able to predict high-stress situations for the surgeon during surgery would allow measures to be taken to reduce their impact on the quality of care.

There are traditional methods to assess the workload, both mental and physical, of surgeons during their surgical activity, such as the subjective SURG-TLX scale [4]. However, the evolution in wearable technology allows us to have sensors of reduced size, which facilitate the recording and analysis of physiological and ergonomic parameters of the surgeon, without interrupting their surgical performance. Some of these objective parameters are the surgeon’s posture by means of body motion techniques, force/torque analysis [5], muscle activity by analyzing the electromyographic (EMG) signal [6,7], and the level of stress by examining the electrocardiogram (ECG) or the electrodermal activity (EDA) signals [8,9].

Recording data on the surgeon’s ergonomics and surgical performance using wearable technology can be a powerful tool for monitoring the surgeon’s health conditions during surgery, as well as the quality of care provided to the patient. Similarly, these technological innovations can offer robust solutions for the analysis and comparison of objective physiological and ergonomic parameters during surgery. 

Therefore, the aim of this study is to implement a set of wearable technologies for the recording and analysis of the surgeon’s physiological and ergonomic parameters during surgical performance in laparoscopic and robotic-assisted surgery. Moreover, different ergonomic and stress factors of surgeons during conventional and robotic-assisted laparoscopic practice will be compared to better identify the improvements and deficiencies that the use of surgical robotics entails in surgical practice.

## 2. Materials and Methods

**Figure 1:**
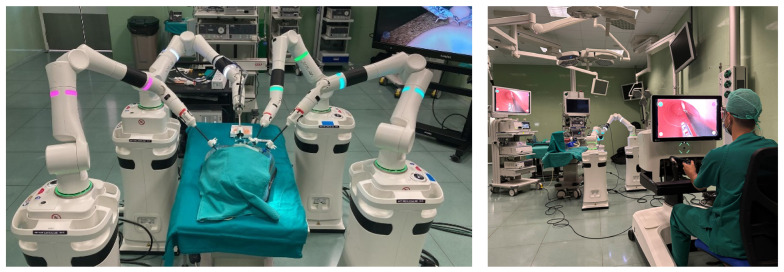
Versius^TM^ Robotic Platform: instrument beside units (**left**) and surgeon console (**right**).

The studies were carried out using standard equipment for conventional laparoscopic surgery and robotic-assisted surgery. Specifically, an Olympus^TM^ Visera III (Tokyo, Japan) tower was used during laparoscopic activities and the Versius^TM^ robotic platform (CMR Surgical; Cambridge, UK) for the robotic-assisted surgical activities (Figure 1).

The studies were organized in two different setups: simulator studies and experimental model studies. All participating surgeons received training sessions in the use of the robotic platform, which included at least two consecutive days practicing with tasks similar to the protocol used during the study. The objective of the training sessions was to learn the basic aspects of the use of the platform, the handling of the controls, and its main functionalities to be able to perform safe surgeries.

To ensure the same ergonomic conditions for all surgeons, in the case of laparoscopic practice, the height of the operating table was adjusted according to the surgeon’s height, and the monitor was placed in front of the surgeon and at eye level. In the case of robotic-assisted practice, the surgeon adjusted the height of the screen and console according to the height of his/her eyes and forearms.

### 2.1. Evaluation in a Simulation Setting

Surgeons with different levels of experience in laparoscopic surgery and no experience in surgical robotics participated in this study. All participating surgeons in this study performed a set of tasks using both conventional laparoscopic surgery and the surgical robotics platform.

Peg transfer. Eye–hand coordination task consisting of transferring rubber pieces in the form of elongated toroids from one pole to another by passing the piece from one hand to the other. Two fenestrated forceps were used for the dominant and non-dominant hands. A repetition was considered when the surgeon moves all three pieces to the three target poles, and the entire task consisted of two repetitions. A time limit of ten minutes was set for this task.

Labyrinth. In this task, participants were asked to pass a needle through a circuit of rings, with different orientations and heights. The needle must be inserted with the dominant hand and withdrawn on the other side with the non-dominant hand. A needle holder is used in the dominant hand and a Maryland dissector in the other hand. The resolution of the entire circuit is considered as a repetition. A time limit of ten minutes is set for this task.

Suture. For this task, participants were asked to make a stitch in an organic tissue (ex vivo pig stomach). The suture consisted of needle passage, a double knot, and two single knots in opposite directions. A needle holder is used in each hand. A time limit of ten minutes was established for suturing.

### 2.2. Evaluation in Experimental Model

The surgeons performed a set of surgical procedures according to their surgical specialty both by conventional laparoscopy and with the robotic platform. A total nephrectomy in porcine model was performed for the urology specialty, a gastrotomy in porcine model for the general surgery specialty, and an ovariectomy in a sheep model for the gynecology specialty. All experimental protocols were approved by the corresponding local Animal Experimentation Committee (References: EXP-20230325, EXP-20230320, and EXP-20230313).

### 2.3. Subjective Evaluation

At the beginning and at the end of each surgical task/procedure, participants were asked to complete a SURG-TLX survey [4]. This questionnaire allows the evaluation of the surgeon’s mental and physical workload before and during the performance of the surgical activities. Among the aspects to be evaluated are mental, physical, and temporal demands, level of perceived stress, distraction, and level of complexity of the task performed.

### 2.4. Data Recording

#### 2.4.1. Physiological Data

Wearable devices (EcgMove 4 and EdaMove 4 activity sensors; Movisens GmbH, Karlsruhe, Germany) were used to record the electrocardiogram (ECG) and electrodermal activity (EDA), respectively, of the participating surgeons. The data recorded from the EDA and ECG sensors were in microsiemens and heart rate, with an output rate of 32 Hz and 1024 Hz, respectively. DataAnalyzer software from Movisens GmbH was used for date analysis.

**Figure 2:**
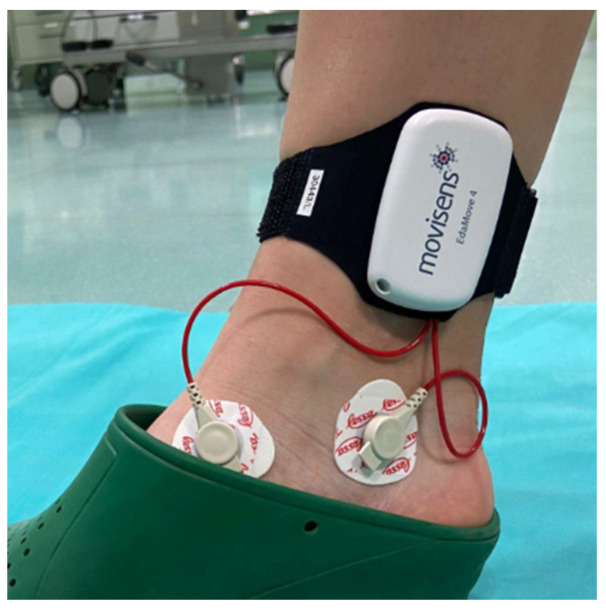
EdaMove 4 activity sensor placed on the surgeon’s ankle.

The EDA sensor was attached to the front of the right ankle of each participant by means of a band (Figure 2) and the ECG sensor was attached on the chest, under the left pectoral, for each participant.

#### 2.4.2. Kinematic Data

**Figure 3:**
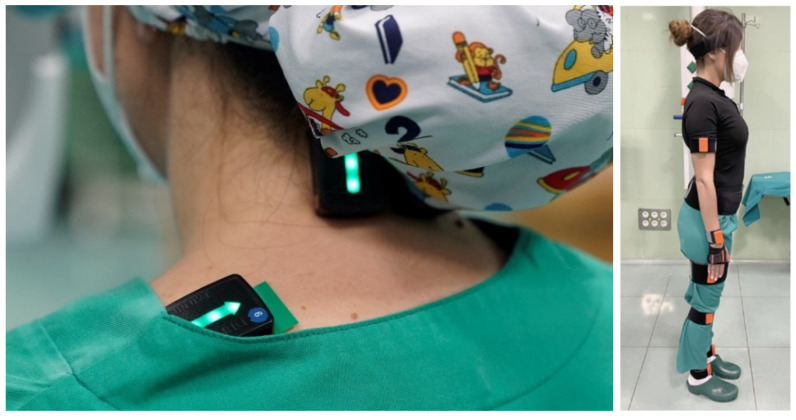
Example of location of EMG sensors (**left**) and inertial sensors for motion analysis (**right**).

The Xsens motion analysis system (Movella Inc.; Henderson, NV, USA) was used to capture the movement of the study participants (Figure 3). This system uses 17 inertial sensors to record the movements of the subject’s body segments in real time, with an update rate up to 60 Hz per sensor.

In addition, the wireless EMG TRIGNO™ Avanti system from DELSYS was used to record the muscle activity by means of electromyographic (EMG) signals (Figure 3). This system has up to 16 sensors with a sampling rate of 1024 Hz. A trigger system was used for recording synchronization between the Xsens and Delsys systems. The EMG signal of following muscle groups was recorded bilaterally: upper trapezius, middle trapezius, erector spinae, brachioradialis, triceps brachii, vastus lateralis, and gastrocnemius medialis. The EMG sensors were placed on each muscle according to the SENIAM guidelines [10]. Prior to the placement of each sensor, the skin was cleaned by gently rubbing it with 70% isopropyl alcohol.

Raw EMG signals were processed using a band-pass filter of 20–300 Hz. The filtered EMG was then smoothed using an algorithm with a moving window of 125 ms and calculated as root mean square (RMS). To normalize the results for each subject, the EMG values were presented as percentage of maximum voluntary contraction (%MVC). MVC was performed separately for each muscle group just before each test by asking each subject to perform specific contractions against a fixed resistance.

### 2.5. Data Analysis

#### 2.5.1. Stress

Mean EDA and ECG sensor values were obtained for each surgeon and for each task or surgical procedure analyzed. The correlation between the parameters reported by the surgeons for the workload levels (SURG-TLX) and the values recorded by the EDA and ECG sensors was also computed.

#### 2.5.2. Motion Analysis

Flexion/extension of the neck, shoulder, elbow, wrist, back, and knee, as well as abduction/adduction of the shoulder, was analyzed for representative joints in the analysis of the surgeon’s posture in robotic-assisted surgical practice [1]. Average values for the degrees of each analyzed joint were obtained and compared between study groups. 

In addition, the surgeon’s body posture was assessed considering the rapid upper limb assessment (RULA) method [11]. RULA gives a score of the posture of the upper and lower limb joints, as well as an overall score of the subject’s posture, establishing a certain level of risk of developing musculoskeletal disorders. 

Other techniques that can be used to provide a body posture score include the rapid entire body assessment (REBA) method [12] or the Ovako working analysis system (OWAS) method [13]. However, the RULA method is optimal for upper-body-intensive tasks such as surgical practice. The REBA method is less detailed in the evaluation of specific postures, as it is mainly recommended for tasks that require rapid changes in body posture, mostly produced by instability and heavy weights. The OWAS method is an observational method that classifies combinations of body postures and is often used as a first approximation but is often complemented by another method (such as RULA or REBA) for a more complete evaluation. 

#### 2.5.3. Localized Muscle Fatigue

For the study of localized muscle fatigue, the evolution of the amplitude and median frequency of the muscle activity was analyzed for each task and surgical procedure. Muscular fatigue was quantified using the joint analysis of spectrum and amplitude (JASA) method [14].

### 2.6. Statistical Analysis

Jamovi (Jamovi Project, 2024, Version 2.5) and R (R Foundation for Statistical Computing, Vienna, Austria; Version 4.3.1) software platforms were used for data and statistical analysis.

## 3. Results

### 3.1. Evaluation in a Simulation Setting

Six surgeons participated in the study in the simulated setting: three experts (>100 laparoscopic procedures performed; 1.80 ± 0.10 m tall and 36.33 ± 13.65 years old) and three novices (<100 laparoscopic procedures performed; 1.82 ± 0.05 meters tall and 29.33 ± 8.39 years old) in laparoscopic surgery, both with no experience in robotic surgery.

The results of the simulator tasks focus mainly on the suturing task, as it is considered the most complete and technically demanding, although the other simulation tasks are also taken into account.

#### 3.1.1. Stress

**Figure 4:**
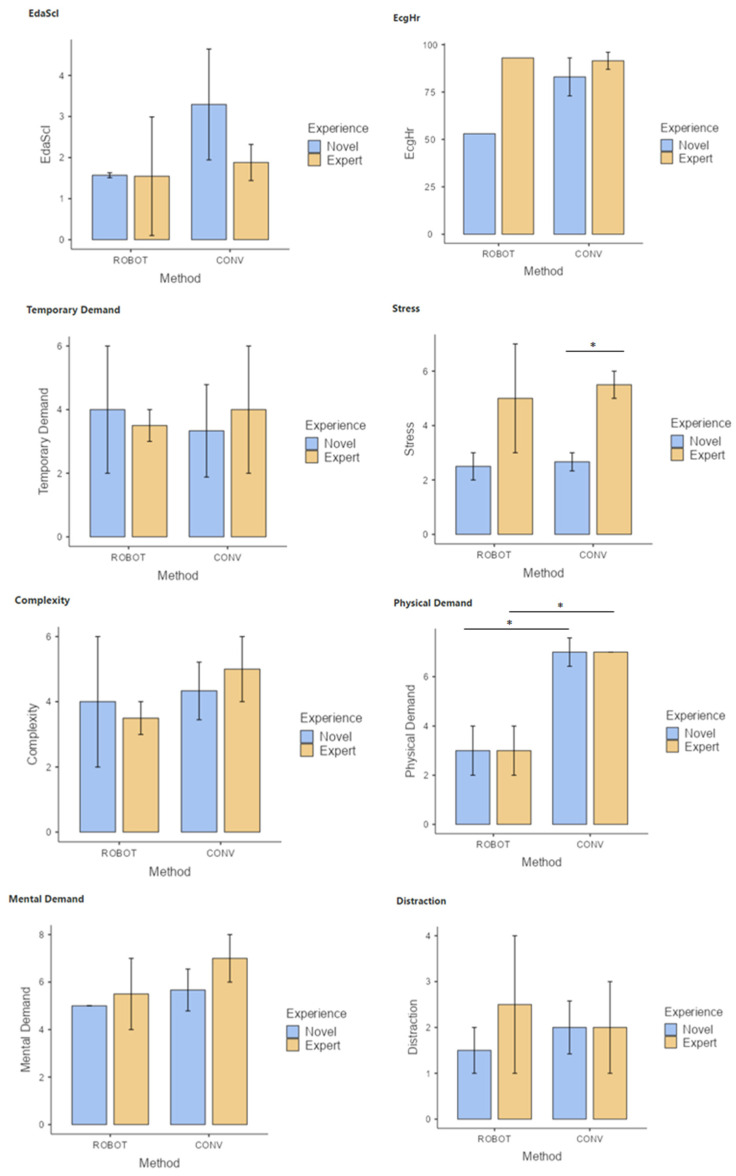
Comparison of SURG-TLX parameters (mental demand, temporal demand, physical demand, stress, task complexity, and distractions), and EDA and ECG signal results during simulator tasks using conventional (CONV) and robotic-assisted (ROBOT) laparoscopy for novice and experienced laparoscopic surgeons. * *p* < 0.05.

Surgeons, both novice and experienced in laparoscopic surgery, report a lower physical demand in the performance of the training tasks using robotic-assisted surgery compared to the conventional laparoscopic technique (Figure 4). Surgeons with experience in laparoscopic surgery reported higher levels of stress in the performance of the tasks using conventional laparoscopic surgery.

A strong positive correlation was observed between the level of stress and the level of complexity of the tasks, as well as between the reported mental demand and the level of distractions, in the group of novice surgeons.

#### 3.1.2. Motion Analysis

**Figure 5:**
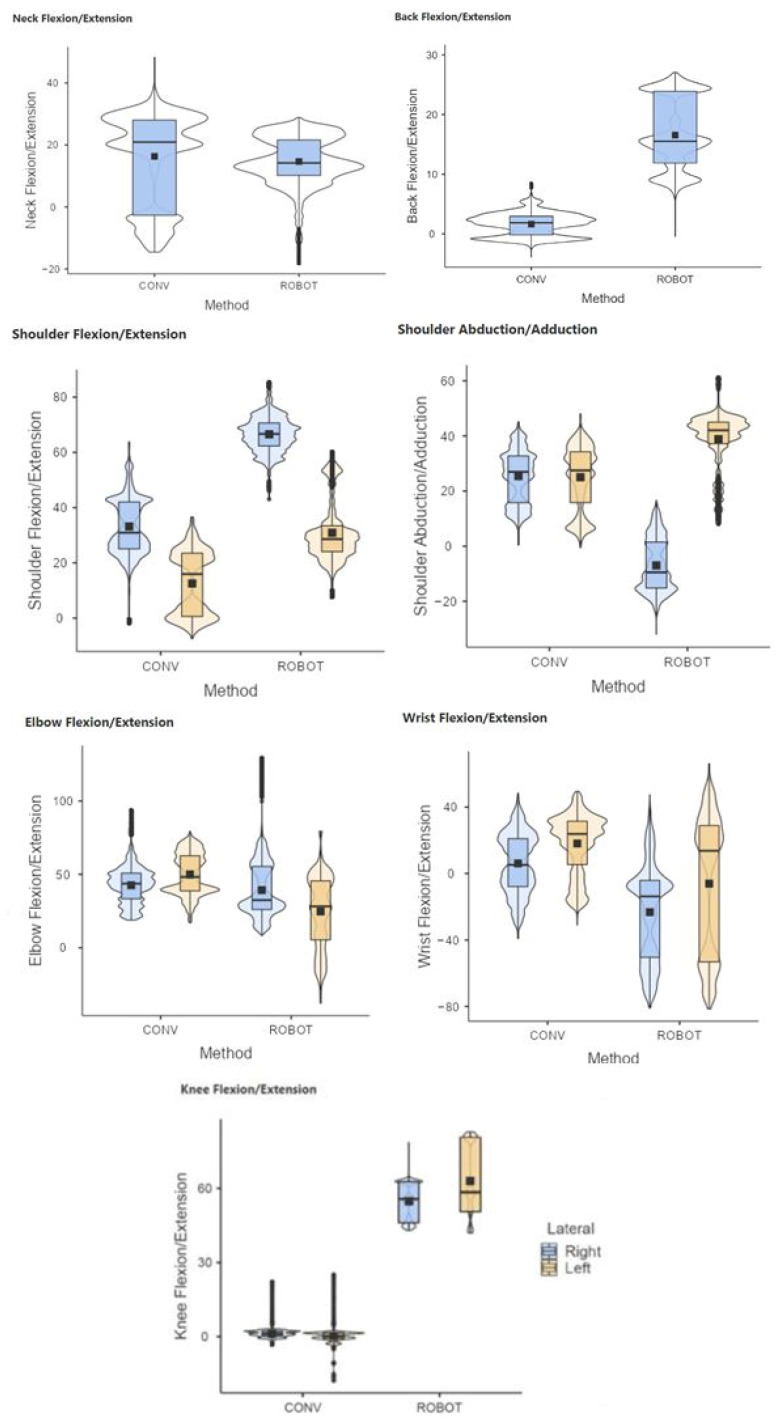
Comparative range of motion of the neck, back, shoulder, elbow, wrist, and knees during laparoscopic (CONV) and robotic-assisted (ROBOT) suture on simulator. Group of novice surgeons in laparoscopic surgery.

**Figure 6:**
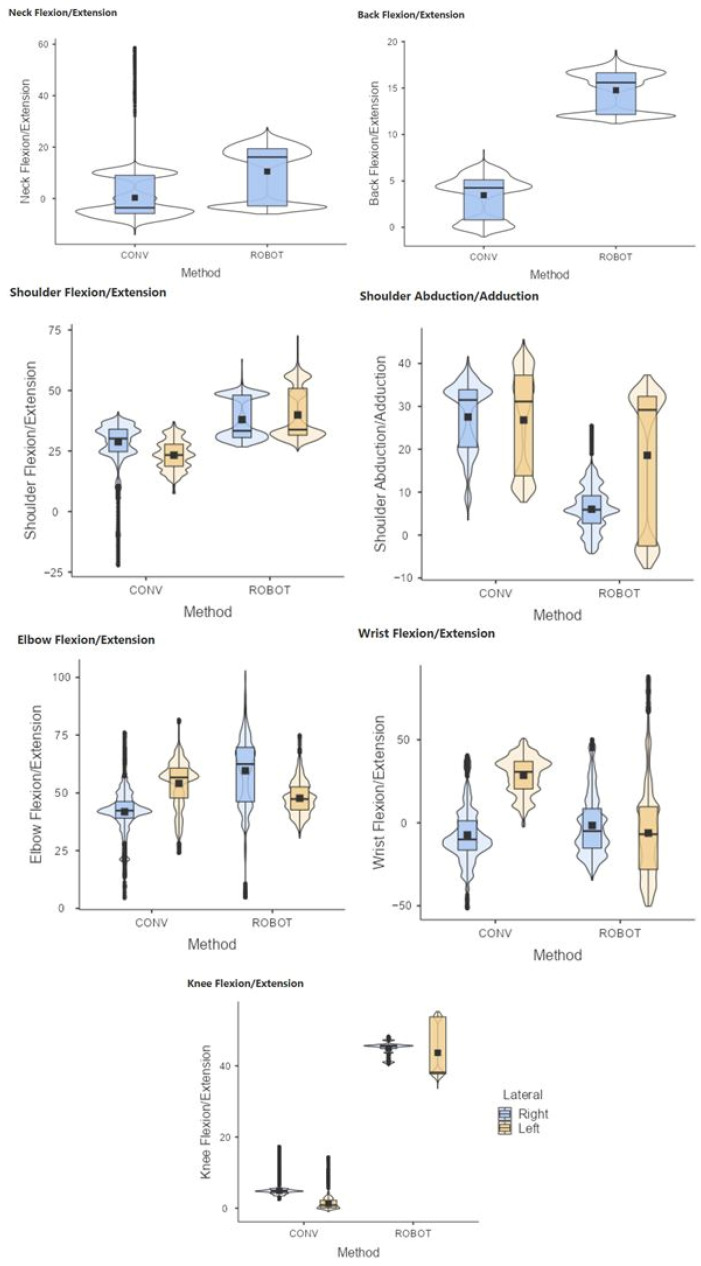
Comparative range of motion of the neck, back, shoulder, elbow, wrist, and knees during laparoscopic (CONV) and robotic-assisted (ROBOT) suture on simulator. Group of experienced surgeons in laparoscopic surgery.

The results showed statistically significant differences (*p* < 0.05) in conventional and robotic-assisted laparoscopic suturing, for both the experienced and novice surgeons, regarding the joint postures analyzed (Figure 5 and Figure 6).

The RULA during the suturing task shows a higher RULA score in the use of the laparoscopic technique compared to the robotic-assisted one (Table 1). The overall score with the laparoscopic technique suggests changes in the surgeon’s posture.

#### 3.1.3. Muscle Activity

**Figure 7:**
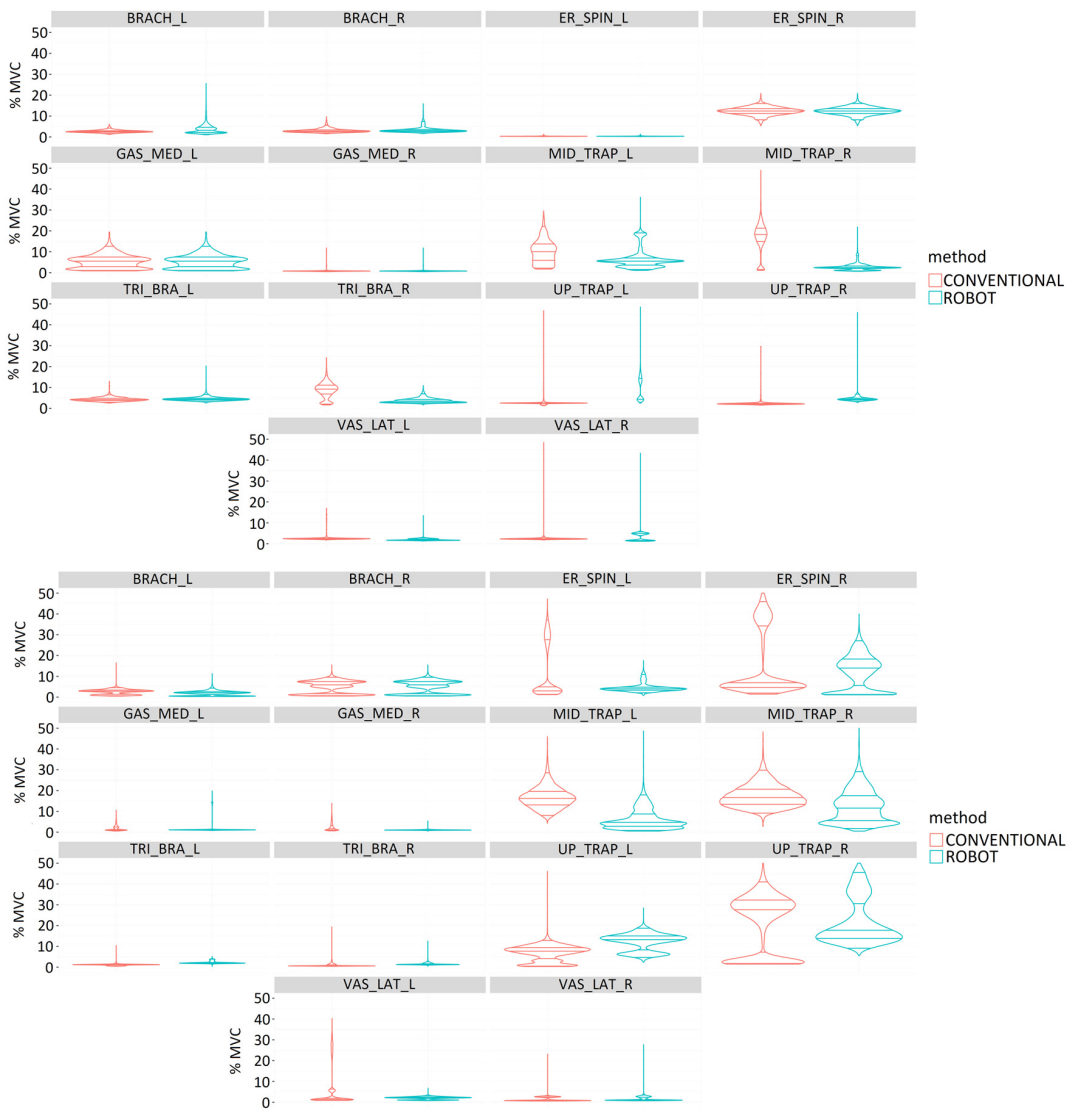
Comparison of muscle activity (%MVC) of experienced (**upper image**) and novice (**bottom image**) surgeons during performance of simulator suturing task using conventional (red) and robotic-assisted (blue) laparoscopic surgery for the following muscles: Brachioradialis (BRACH), Erector spinae (ER_SPIN), Gastrocnemius medialis (GAS_MED), Middle trapezius (MID_TRAP), Triceps brachii (TRI_BRA), Upper trapezius (UP_TRAP), and Vastus lateralis (VAS_LAT).

In general, experienced surgeons had less muscle activity in the right-sided muscles (Figure 7, upper graph). In laparoscopic surgery, they required greater muscle activity of the middle trapezius and triceps brachii on the right side than with robotic-assisted surgery. Robotic-assisted surgery required greater muscle activity for the brachioradialis and upper trapezius bilaterally, left middle trapezius, and right vastus lateralis.

For novice surgeons (Figure 7, bottom graph), robotic-assisted surgery required greater muscle activity for the upper trapezius and vastus lateralis on the right side and the gastrocnemius medialis on the left side. Laparoscopic surgery required greater muscle activity of the erector spinae and middle trapezius bilaterally, the right triceps brachii and gastrocnemius medialis, and the left upper trapezius and vastus lateralis. All muscles bilaterally showed statistically significant differences between laparoscopic and robotic-assisted surgery (*p* < 0.001).

For the remaining simulator tasks, labyrinth and peg transfer, the results obtained resembled those of the suturing task. Thus, these results also recorded higher muscle activity on the left side.

#### 3.1.4. Localized Muscle Fatigue

**Figure 8:**
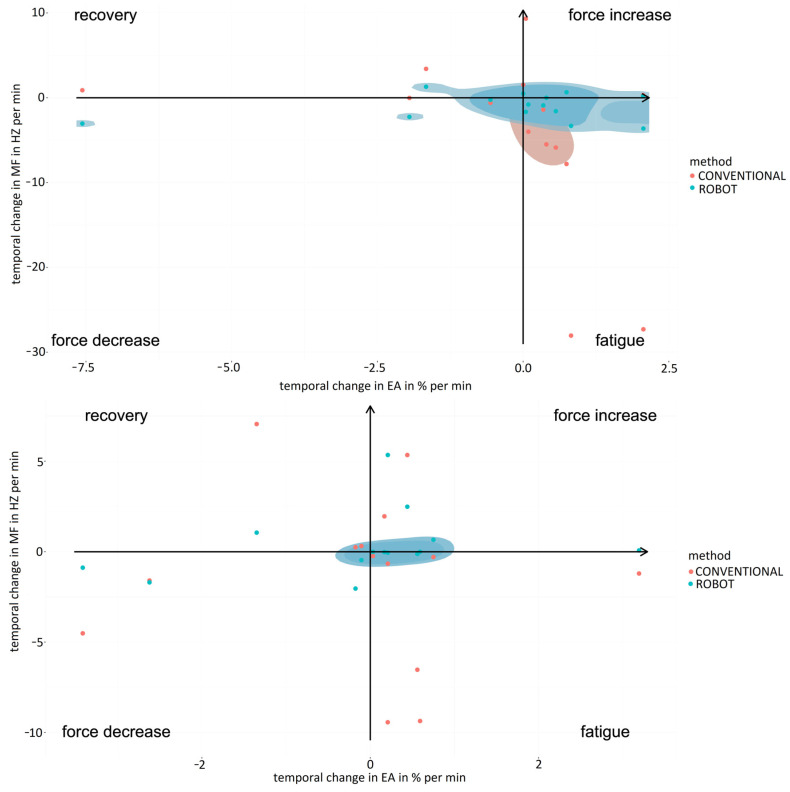
Comparison of fatigue and muscle strength increase/decrease for experienced (**upper graph**) and novice (**bottom graph**) surgeons between simulator suturing task in laparoscopic (red) and robotic-assisted (blue) surgeries.

Analysis of localized muscle fatigue during the simulator suturing task showed a trend of higher muscle fatigue and lower force use for laparoscopic surgery compared to robotic-assisted surgery for the expert surgeons (Figure 8, upper graph). These results were similar to those obtained for the labyrinth and peg transfer tasks, highlighting that for peg transfer robotic-assisted surgery shows a high degree of muscle fatigue recovery and lower muscle fatigue. The mean values obtained for the percentage of force increase were 4.31 ± 2.35 in the case of the conventional laparoscopic surgery and 0.98 ± 0.76 for the robotic-assisted laparoscopic surgery. In the case of the percentage of muscle fatigue, the values obtained were 11.44 ± 8.27 and 2.11 ± 1.37, respectively, for the conventional and robotic-assisted laparoscopic surgery. For the percentage of muscle fatigue recovery, for conventional laparoscopic surgery the mean value was 2.12 ± 0.31 and for the robotic-assisted laparoscopic surgery the value was 5.70 ± 2.75. For the percentage of force decrement, for the conventional laparoscopic surgery the value obtained was 3.92 ± 2.85 and for the robotic-assisted laparoscopic surgery the value was 1.28 ± 0.81.

For novice surgeons (Figure 8, bottom graph), the simulator suturing task showed markedly greater muscle fatigue for laparoscopic surgery compared to robotic-assisted surgery. However, for the labyrinth task, robotic-assisted surgery showed a high degree of muscle fatigue recovery and a lower degree of force use compared to laparoscopic surgery. Robotic-assisted surgery was more balanced in terms of muscle fatigue and force use than laparoscopic surgery for both experienced and novice surgeons. The mean values obtained for the percentage of force increase were 3.66 ± 2.41 in the case of the conventional laparoscopic surgery and 3.01 ± 1.80 for the robotic-assisted laparoscopic surgery. In the case of the percentage of muscle fatigue, the values obtained were 4.36 ± 3.08 and 0.31 ± 0.23, respectively, for the conventional and robotic-assisted laparoscopic surgeries. For the percentage of muscle fatigue recovery, for conventional laparoscopic surgery the mean value was 2.60 ± 1.71 and for the robotic-assisted laparoscopic surgery the value was 3.97 ± 0.41. For the percentage of force decrement, for the conventional laparoscopic the value obtained was 4.35 ± 1.83 and for the robotic-assisted laparoscopic the value was 2.29 ± 1.36.

**Figure 9:**
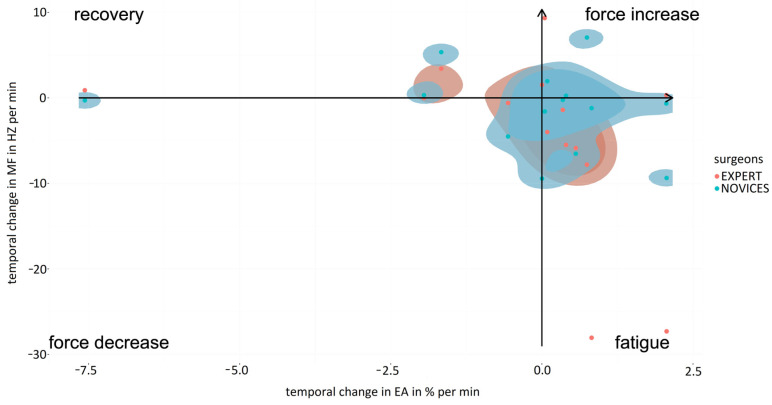

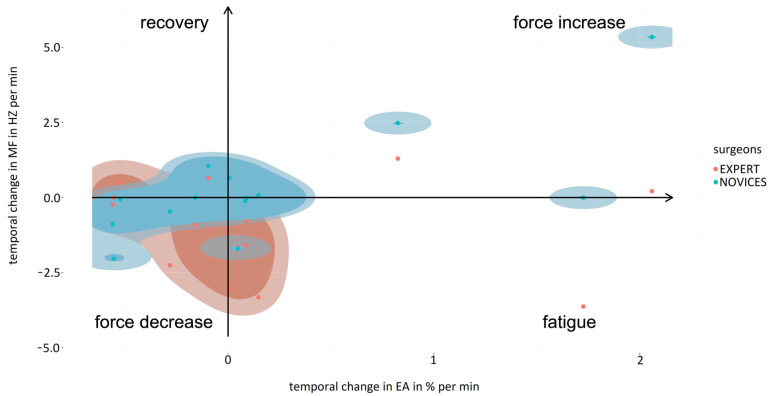
Comparison of fatigue and force increasing/decreasing when performing suturing task in robotic-assisted (**upper graph**) and laparoscopic surgeries (**bottom graph**) between expert surgeons (blue) and novice surgeons (red).

In the case of robotic-assisted surgery, localized muscle fatigue during the simulator suturing task was similar for expert and novice surgeons (Figure 9, upper graph). However, expert surgeons used more force than novices. Consequently, the mean values obtained for the percentage of force increase were 2.83 ± 1.79 in the case of the expert surgeon and 1.80 ± 0.37 for the novice surgeons. In the case of the percentage of muscle fatigue, the values obtained were 2.40 ± 1.23 and 0.86 ± 0.60, respectively, for the expert and novice surgeons. For the percentage of muscle fatigue recovery, for novice surgeons the mean value was 1.06 ± 0.06 and for the expert surgeons the value was 0.64 ± 0.08. For the percentage of force decrement, for the expert surgeons the value obtained was 1.27 ± 0.88 and for the novice surgeons the value was 0.88 ± 0.61. In the case of laparoscopic surgery, localized muscle fatigue during the simulator suturing task showed a trend of higher muscle fatigue and lower use of force for expert surgeons compared to novices (Figure 9, bottom graph). Of note was the muscle fatigue of the novice surgeons and the near-zero use of force by the expert surgeons. On the other hand, expert surgeons were more balanced in terms of muscle fatigue and use of force than novice surgeons. The mean values obtained for the percentage of force increase were 4.31 ± 3.35 in the case of the expert surgeons and 3.17 ± 2.48 for the novice surgeons. In the case of the percentage of muscle fatigue, the values obtained were 11.45 ± 9.29 and 4.46 ± 1.97, respectively, for the expert and novice surgeons. For the percentage of muscle fatigue recovery, for novice surgeons the mean value was 5.70 ± 2.70 and for the expert surgeons the value was 3.78 ± 1.55. For the percentage of force decrement, for the expert surgeons the value obtained was 6.06 ± 2.13 and for the novice surgeons the value was 1.28 ± 0.71. These results were similar to those obtained for the labyrinth and transfer peg tasks.

### 3.2. Evaluation in Experimental Model

Three surgeons experienced in laparoscopic surgery (>100 laparoscopic procedures performed; 1.77 ± 0.07 m tall and 37.00 ± 5.29 years old) and novices in robotic surgery participated in the study with experimental models. Each surgeon performed the surgical procedures according to his/her specialty: urology, gynecology, or general surgery.

#### 3.2.1. Stress

Surgeons presented higher levels of electrodermal activity during robotic-assisted procedures. Similarly, the reported levels of stress, mental demand, and task complexity were higher in surgical robotics compared to the conventional laparoscopic technique. However, the reported physical demand was higher in the case of laparoscopic procedures. Although remarkable, no statistically significant differences were shown in these results.

A positive correlation has been observed between the temporal demand of the surgical procedure and the physical demand (0.868 Spearman’s rho; *p* < 0.05) and complexity (0.893 Spearman’s rho; *p* < 0.02) reported by surgeons, as well as between the subjective stress during surgical interventions and distraction perceived (0.890 Spearman’s rho; *p* < 0.02).

#### 3.2.2. Motion Analysis

**Figure 10:**
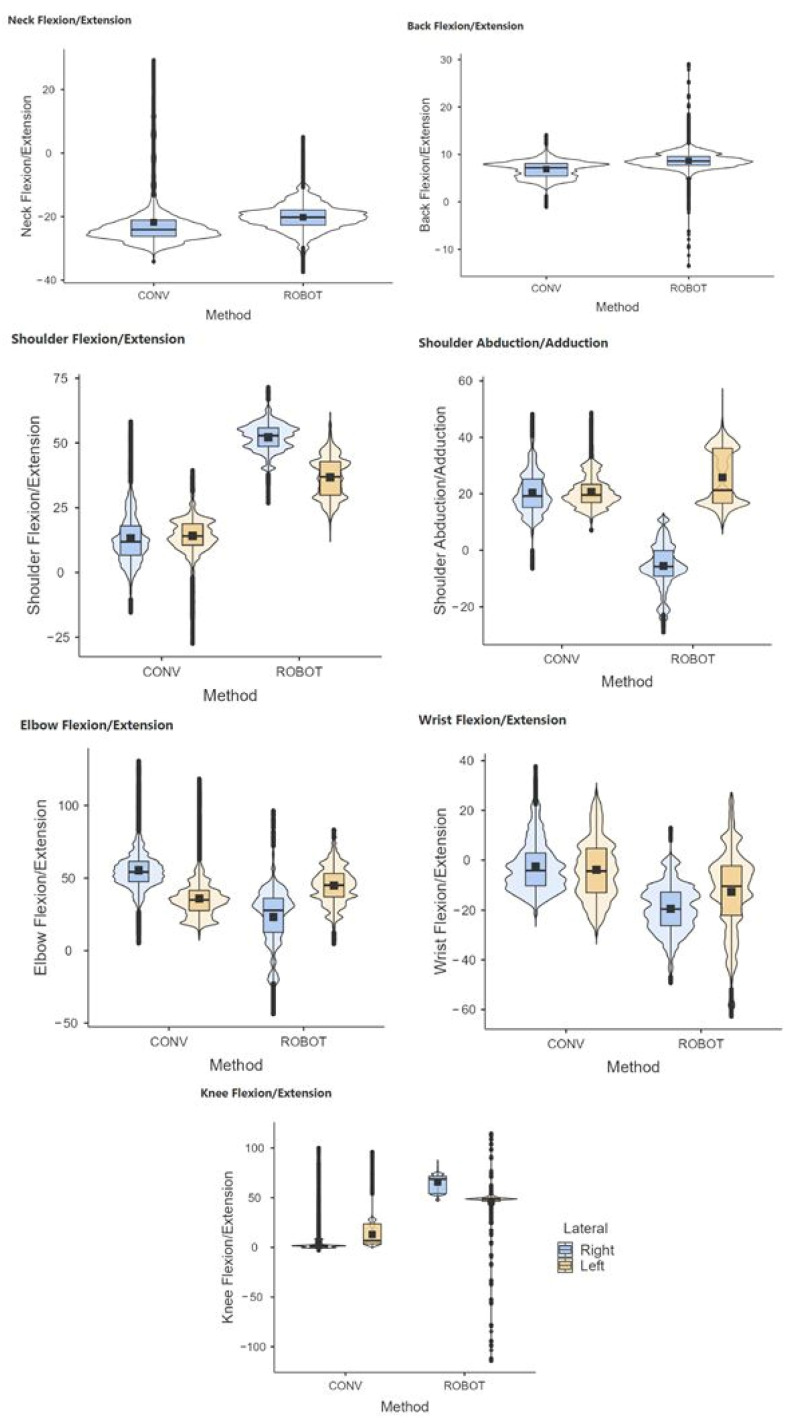
Comparative range of motion of the neck, back, shoulder, elbow, wrist, and knees during conventional (CONV) and robotic-assisted (ROBOT) laparoscopic gastrotomy.

**Figure 11:**
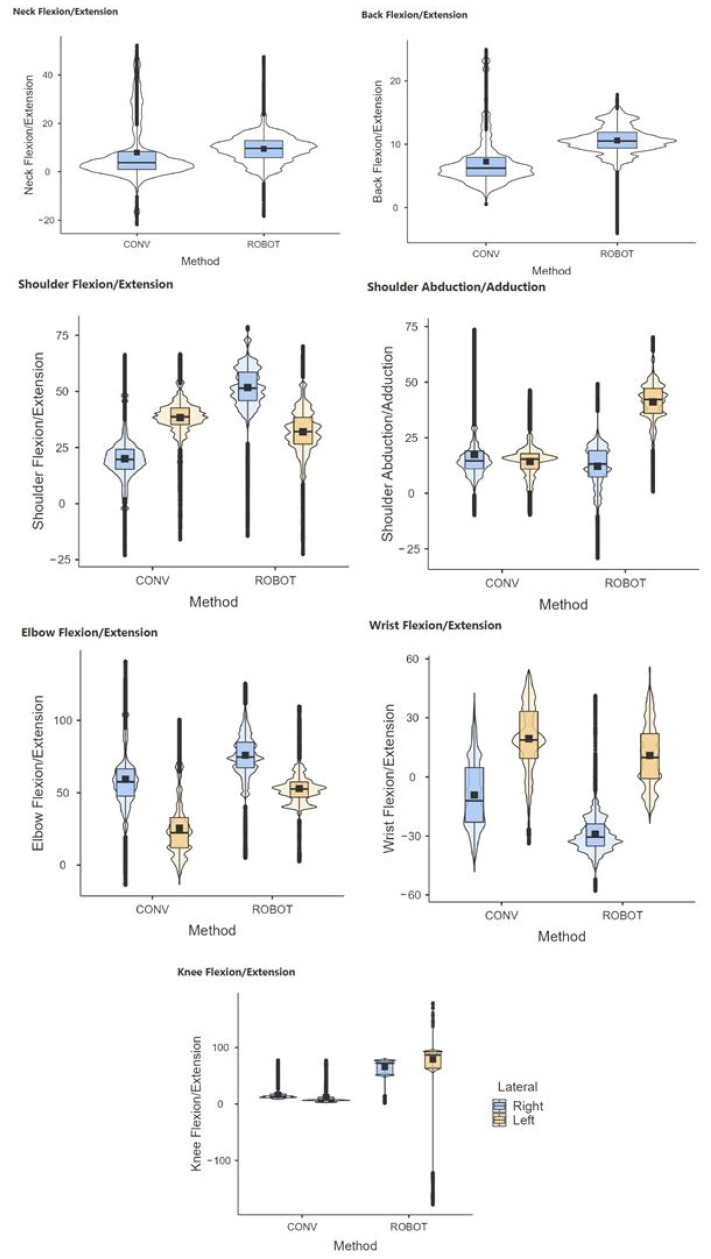
Comparative range of motion of the neck, back, shoulder, elbow, wrist, and knees during conventional (CONV) and robotic-assisted (ROBOT) laparoscopic total nephrectomy.

**Figure 12:**
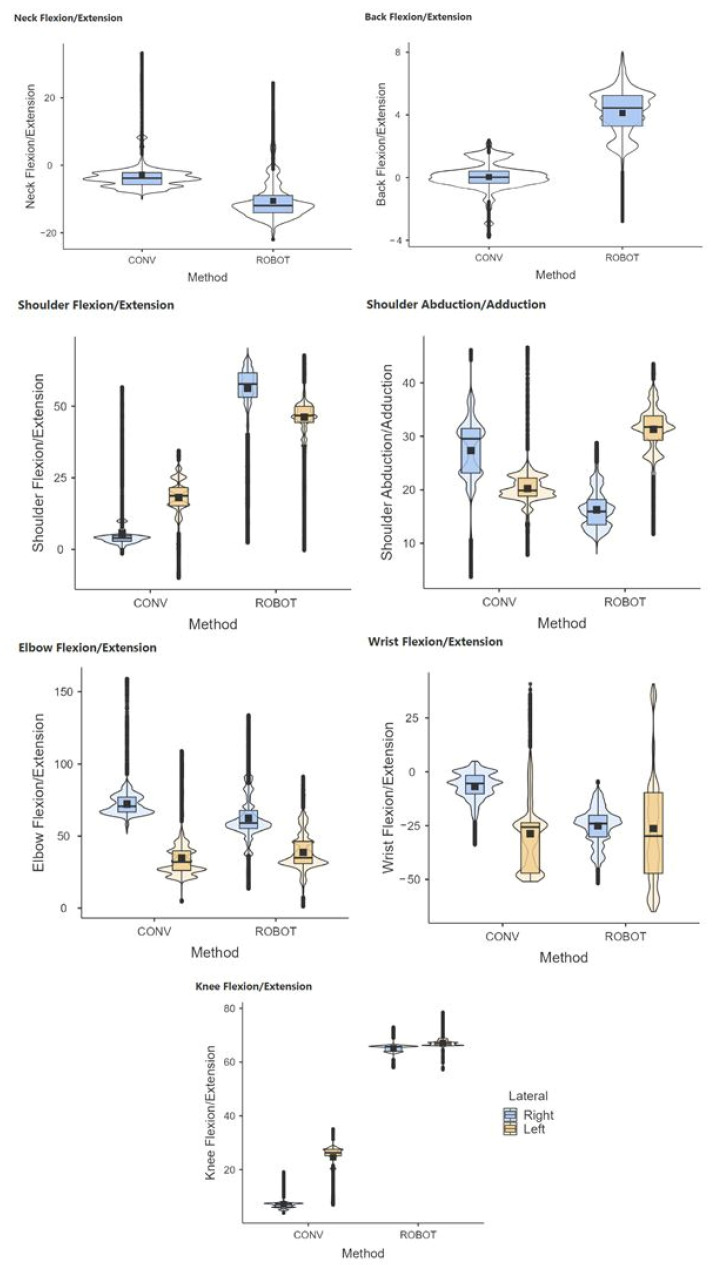
Comparative range of motion of the neck, back, shoulder, elbow, wrist, and knees during conventional (CONV) and robotic-assisted (ROBOT) laparoscopic total ovariectomy.

The results showed statistically significant differences (*p* < 0.05) in conventional and robotic-assisted laparoscopic procedures for the joint postures analyzed, except for the shoulder during the ovariectomy (Figure 10, Figure 11 and Figure 12).

Analyzing the ergonomic risk level of the surgeon’s posture, the surgeon’s posture presents a low level of ergonomic risk during the performance of total nephrectomy, both by conventional laparoscopic and robotic-assisted surgeries (Table 2). In the case of gastrotomy, it presents a medium level of risk for both techniques, so it is recommended to improve the surgeon’s posture as soon as possible. Finally, the ergonomic risk level of the surgeon’s posture was medium for ovariectomy performed by conventional laparoscopic surgery, however, it obtained a high risk level during robotic-assisted ovariectomy, so it is recommended that the surgeon’s posture be improved immediately.

#### 3.2.3. Muscle Activity

**Figure 13:**
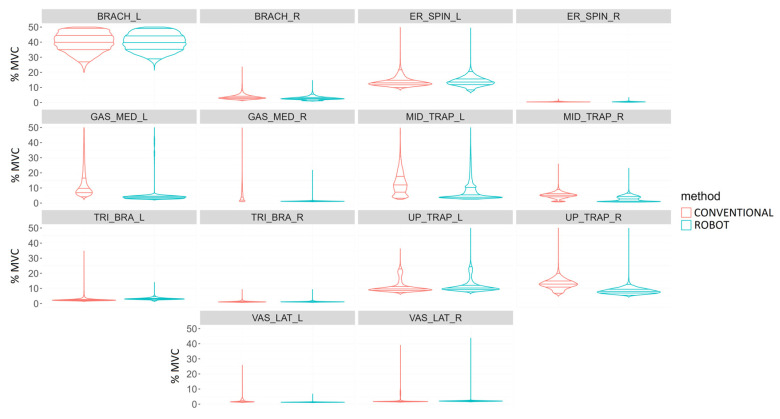
Comparison of muscle activity (%MVC) during the performance of a gastrotomy by conventional (red) and robotic-assisted (blue) laparoscopic surgeries for the following muscles: Brachioradialis (BRACH), Erector spinae (ER_SPIN), Gastrocnemius medialis (GAS_MED), Middle trapezius (MID_TRAP), Triceps brachii (TRI_BRA), Upper trapezius (UP_TRAP), and Vastus lateralis (VAS_LAT).

In general, the muscles on the right side recorded lower muscle activity during gastrotomy (Figure 13). Robotic-assisted surgeries required greater muscle activity for the right vastus lateralis. The laparoscopic procedure required higher muscle activity for the gastrocnemius medialis and middle trapezius bilaterally, the right brachioradialis and upper trapezius superiorly, and the left triceps brachii and vastus lateralis. All muscles bilaterally showed statistically significant differences between laparoscopic and robotic-assisted surgeries (*p* < 0.001).

**Figure 14:**
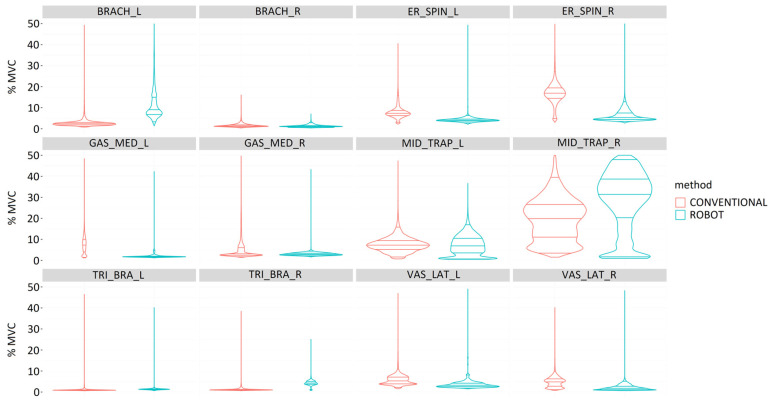
Comparison of muscle activity (%MVC) during the performance of a total nephrectomy by conventional (red) and robotic-assisted (blue) laparoscopic surgeries for the following muscles: Brachioradialis (BRACH), Erector spinae (ER_SPIN), Gastrocnemius medialis (GAS_MED), Middle trapezius (MID_TRAP), Triceps brachii (TRI_BRA), Upper trapezius (UP_TRAP), and Vastus lateralis (VAS_LAT).

During the performance of total nephrectomy, muscle activity was generally more balanced between the right and left sides compared to other surgical procedures (Figure 14). In this surgical procedure, robotic-assisted surgery required greater muscle activity of the left brachioradialis and of the middle trapezius, triceps brachii, and vastus lateralis on the right side. Surgeries with conventional laparoscopic technique led to greater muscle loading of the erector spinae and gastrocnemius medialis bilaterally, middle trapezius, triceps brachii, and vastus lateralis on the left side. All bilateral muscles showed statistically significant differences between laparoscopic and robotic-assisted surgeries (*p* < 0.001).

**Figure 15:**
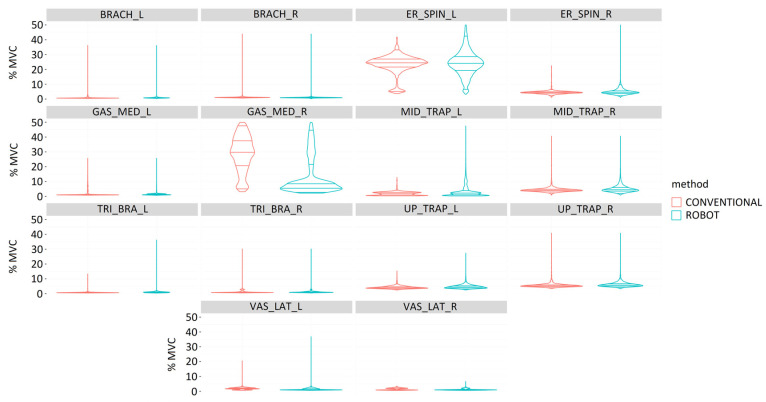
Comparison of muscle activity (%MVC) during the performance of an ovariectomy by conventional (red) and robotic-assisted (blue) laparoscopic surgeries for the following muscles: Brachioradialis (BRACH), Erector spinae (ER_SPIN), Gastrocnemius medialis (GAS_MED), Middle trapezius (MID_TRAP), Triceps brachii (TRI_BRA), Upper trapezius (UP_TRAP), and Vastus lateralis (VAS_LAT).

With respect to ovariectomy, in general, the muscles on the right side recorded slightly higher muscle activity than the muscles on the left side (Figure 15). Conventional laparoscopic surgery required greater activity of the gastrocnemius medialis and vastus lateralis on the right side. Robotic-assisted surgery required greater muscle activity for the right erector spinae and the middle and upper trapezius, triceps brachii, and vastus lateralis on the left side. All bilateral muscles showed statistically significant differences between laparoscopic and robotic-assisted surgeries (*p* < 0.001).

#### 3.2.4. Localized Muscle Fatigue

**Figure 16:**
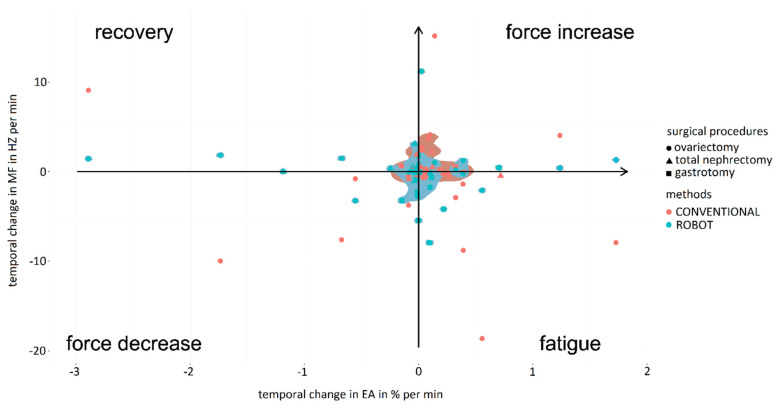
Comparison of fatigue and increase/decrease in force exerted by surgeons during the performance of surgical procedures (circle: ovariectomy; triangle: total nephrectomy; square: gastrotomy) using conventional (red) and robotic-assisted (blue) laparoscopic surgeries.

The analysis of localized muscle fatigue during different surgical procedures showed lower muscle fatigue and higher force use in laparoscopic procedures compared to robotic-assisted ones (Figure 16). However, robotic-assisted procedures reported more balanced muscle fatigue and force use than laparoscopic ones. Considering surgical specialties, the results for ovariectomy were very scattered, occupying the extremes for localized muscle fatigue and force use for both surgical techniques. Consequently, the mean values obtained for the percentage of force increase were 6.47 ± 5.85 in the case of the conventional laparoscopic surgery and 4.10 ± 2.89 for the robotic-assisted laparoscopic surgery. In the case of the percentage of muscle fatigue, the values obtained were 7.99 ± 6.75 and 1.04 ± 0.97, respectively, for the conventional and robotic-assisted laparoscopic surgeries. For the percentage of muscle fatigue recovery, for conventional laparoscopic surgery the mean value was 2.45 ± 0.53 and for the robotic-assisted laparoscopic surgery the value was 9.52 ± 0.81. For the percentage of force decrement, for the conventional laparoscopic surgery the value obtained was 5.62 ± 3.06 and for the robotic-assisted laparoscopic surgery the value was 2.26 ± 1.71. For total nephrectomy and gastrotomy, the results are more balanced and follow a general trend. In this case, procedures by conventional laparoscopy required high force use and lower localized muscle fatigue. In this way, for total nephrectomy, the mean values obtained for the percentage of force increase were 1.70 ± 1.52 in the case of the conventional laparoscopic surgery and 0.17 ± 0.06 for the robotic-assisted laparoscopic surgery. In the case of the percentage of muscle fatigue, the values obtained were 0.45 ± 0.36 and 0.19 ± 0.08, respectively, for the conventional and robotic-assisted laparoscopic surgery. For the percentage of muscle fatigue recovery, for conventional laparoscopic surgery the mean value was 0.72 ± 0.56 and for the robotic-assisted laparoscopic surgery the value was 1.20 ± 0.81. For the percentage of force decrement, for the conventional laparoscopic surgery the value obtained was 0.62 ± 0.48 and for the robotic-assisted laparoscopic surgery the value was 0.55 ± 0.22. For the gastrotomy, the mean values obtained for the percentage of force increase were 1.13 ± 0.71 in the case of the conventional laparoscopic surgery and 0.88 ± 0.61 for the robotic-assisted laparoscopic surgery. In the case of the percentage of muscle fatigue, the values obtained were 3.23 ± 1.91 and 0.42 ± 0.19, respectively, for the conventional and robotic-assisted laparoscopic surgeries. For the percentage of muscle fatigue recovery, for conventional laparoscopic surgery the mean value was 0.05 ± 0.03 and for the robotic-assisted laparoscopic surgery the value was 0.39 ± 0.08. For the percentage of force decrement, for the conventional laparoscopic surgery the value obtained was 2.89 ± 1.52 and for the robotic-assisted laparoscopic surgery the value was 0.65 ± 0.44.

## 4. Discussion

With the introduction of new robotic systems, many of the ergonomic challenges faced in open and laparoscopic surgery have been overcome, while new ones have been created. Among these, we will highlight the new designs of robotic instrument controls, the use of foot pedals, and the various types of screens (closed, open, and semi-open). Surveys reported that 56.1% of regularly practicing robotic surgeons continue to experience related physical symptoms or discomfort, mainly neck stiffness and finger and eye fatigue [15].

Analyzing surgeon stress levels during laparoscopic and robotic-assisted surgeries is a critical task since surgeon stress can directly affect surgeon performance and decision-making ability during surgery. High levels of stress can lead to errors, lengthen operative time, and compromise patient safety [16,17]. In addition, stress affects fine motor skills and precision, which are critical in minimally invasive procedures. Stressed surgeons may have difficulty with delicate movements, which can impact surgical outcomes. In addition, prolonged stress can lead to burnout and negatively influence the longevity of a surgeon’s career. Therefore, managing surgeon stress levels helps to improve surgical outcomes, patient safety, and the overall well-being of the surgical team.

A study with the Senhance robotic surgical system (Asensus Surgical, Durham, NC, USA) analyzed the results of the SURG-TLX survey of six centers, covering several disciplines and surgical procedures, both by laparoscopic and robotic-assisted surgeries [16]. Their results showed significantly lower overall workload levels for robotic surgeons compared to laparoscopic surgeons. In general, a higher mental workload was observed in robotic-assisted surgery, but lower physical demands and less distraction. In our study, surgeons also reported a lower physical demand during surgical robotics in both evaluation settings, with a significant difference in the simulation setting. It was observed that this physical demand was strongly influenced by tasks and procedures requiring high temporal demand during surgical procedures and high complexity for simulator tasks. In general, subjective stress was higher for experts than for novice surgeons, being significantly correlated with heart rate (ECG) in laparoscopic tasks. However, in robotic-assisted surgical procedures the level of stress was highly correlated with complexity, mental demand, and electrodermal activity (EDA).

In the case of objective analysis, a study by Sujka et al. demonstrated that robotic surgery could lead to a lower increase in salivary alpha-amylase and cortisol in the surgeon compared with laparoscopic surgery, suggesting a reduction in physiologic stress [17]. For this study, saliva was collected using a passive drool collection system at the beginning, middle, and end of each case; amylase and cortisol were measured by ELISA. In the present study, EDA and ECG were recorded, seeking to achieve physiological data for stress. EDA was higher during laparoscopic procedures, being significant for novice surgeons, while ECG showed a higher level for experts, correlated with their subjective stress. During the surgical procedures the objective and subjective stresses were similar, showing higher EDA during the robotic-assisted surgery. This may be due to the lack of experience with this technique considering that all surgeons had experience in laparoscopic surgery but not in robotic-assisted surgery.

Surgeons experienced increased complexity during conventional laparoscopic interventions, as well as their physical demand or even the objective stress of EDA. This may be because the robotic surgical platform provides easier rotation of surgical instruments, as well as a clutching mechanism that allows them to adapt the position of the instruments during the surgical activity, which improves their ergonomic conditions and enables them to reach remote areas with less effort.

The final objective of every task, the safety of the patient, is tightly related to the complexity and temporary demand experienced by surgeons. Attending to the results from the experimental model, surgeons felt greater subjective stress, task complexity, and mental demand, as well as objective stress from EDA, during the least experienced methodology, resulting in an increase in failure. Similarly, during the simulator model, complexity of the task and errors were also highly correlated with stress.

Surgeon’s motion analysis plays a crucial role in ergonomic analysis within the context of surgery. Ergonomic guidelines for robotic surgery can be significantly improved by considering motion analysis. Understanding how surgeons move during procedures allows for better arrangement of operating rooms and leads to a reduction in mental and physical stress.

The methods for analyzing the surgeon’s posture have evolved dramatically, from methods based on photogrammetry [18], through infrared camera systems in combination with retro-reflective markers [19], which are very limited by occlusions and therefore not suitable for loaded environments such as an operating room. Another study made use of the Xbox Connect camera [20]. This is a video-game-oriented camera that allows measuring the positions of the surgeon’s head, shoulders, mid-spine, hips, and knees using Kinetisense software. However, the system’s accuracy and problems with occlusions do not make it a suitable solution for clinical settings. As a technological evolution, we have inertial sensors, which allow us to record the surgeon’s posture accurately and without limitations of occlusions of the environment. Therefore, this type of system, such as the one used in this study, would certainly be the most appropriate for postural ergonomics analysis in surgical environments.

Within laparoscopic tasks, it is interesting to mention laparoscopic suturing. During conventional laparoscopic surgery, which includes limited degrees of freedom or a two-dimensional view, novice surgeons experience limitations in the learning curve of intracorporeal suturing, whereas robotic-assisted surgery offers benefits in suturing performance and decreases workflow [21].

It should be noted that, in general, the use of the robotic platform leads to greater flexion of the back compared to the conventional laparoscopic technique. However, the flexion is usually less than 15 degrees and therefore not highly ergonomically detrimental [1]. This slight flexion is related to the fact that during robotic-assisted procedures the surgeon remains seated.

During the evaluation of the shoulder posture, we can observe some remarkable behaviors in terms of its abduction/adduction. When surgeons perform tasks with conventional laparoscopy, they must maintain a slight abduction due to the height restrictions of the operating table. However, the robotic platform offers the possibility to clutch the instruments in search of a more ergonomic posture, which leads them to perform the most complex movements with the dominant hand.

Considering that back flexion/extension refers to the lumbar region, the height of the console screen would primarily affect neck posture. The height of the console and the armrests of the robotic platform are synchronized, both adjusting to the elbows so that at rest they should describe 90 degrees. When the surgeon makes this adjustment, the lumbar region is usually adequately positioned in the back of the chair, as we can see in the graphs showing the posture during robot-assisted surgery, with the elbows close to 90º and the back slightly flexed. In conventional laparoscopy, the height of the operating table in an excessively low position can cause the surgeon’s back to bend excessively in order to reach the target anatomical areas.

Analyzing knee flexion/extension in robotic-assisted procedures, some values outside the expected range have been observed for the left knee. It appears that surgeons tend to extend the leg for long periods while sitting to seek rest. This is also a common behavior during driving, mainly dominated by the use of the right foot. On the other hand, some graphs show some negative values (outliers) in knee flexion/extension, which is considered an inconsistent value. This may be due to the calculation performed by the Xsens motion analysis system software. Usually, the posture is calculated considering the relationship between sensors placed on different joint segments (in this case tibia and femur). Perhaps the out-of-range value occurs when the surgeon lifts the knee sufficiently while seated, and therefore the software detects the tibia sensor above the femur sensor. This type of situation needs to be addressed in subsequent studies.

Similar to other studies [20], the RULA values obtained for the surgeon’s posture during robotic practice indicate a medium ergonomic risk. However, the level of ergonomic risk of the surgeon’s posture during robotic-assisted laparoscopic ovariectomy was considered high, which leads to the recommendation that the surgeon should improve his posture as soon as possible. This result is related to a worse ergonomically inadequate upper limb position during surgery. In the case of the simulator tasks, novice surgeons showed a significant improvement in the ergonomics of their posture during robotic-assisted surgery compared to conventional laparoscopic surgery.

One of the advantages of robot-assisted surgery compared to conventional laparoscopic surgery is the adjustment possibilities offered by the platform with respect to the surgeon’s physical characteristics, allowing the height and proximity of the monitor to be adapted, as well as the height of the controls and the armrests. This favors surgeons to have more ergonomically adequate postures during the surgical practice, mainly of the back and neck.

Not only is it important to analyze the posture of the main surgeon, but the rest of the surgical team also plays a critical role in the safety and quality of patient care. In fact, studies show that the main surgeon’s posture at the console of the robotic platform is more ergonomic than that of the assistant at the patient’s bedside [22]. However, as we have seen in the results of this and other studies, the console can also limit posture, increasing static workload that could be associated with musculoskeletal symptoms.

The introduction of robotics into surgical practice requires knowledge and analysis of the surgeon’s muscle activity to ensure proper ergonomics, as well as to guarantee optimal use of robotic instruments, which translates into precise movements during surgery. A study comparing robotic-assisted laparoscopic surgery and conventional laparoscopic surgery found differences in muscle activation patterns [23]. In general, robotic-assisted surgery requires lower levels of muscle activation in the neck and shoulder region. In the present study, significantly higher muscle activation values were obtained in the middle trapezius during robotic-assisted surgery, both in the simulator studies and in the performance of gastrotomy. Similar results regarding elevated muscle activity in the neck muscles in laparoscopic surgery were also presented by Dalsgaard et al. during a comparative study on hysterectomy [24]. In addition, results have shown that when performing more complex and longer surgical procedures, such as gastrotomy, nephrectomy, or ovariectomy, a significant increase in muscle loading on the gastrocnemius medialis is shown during laparoscopic practice compared to robotic-assisted surgery. This may be due to the fact that the surgeon is standing during the surgical activity.

Evidence has been obtained indicating that forearm muscles show a high strain during robotic-assisted surgery, especially those controlling ulnar deviation movements [23]. This result is in line with the present study, which showed a higher muscle activation in the brachioradialis compared to the conventional laparoscopic technique, mainly in the group of surgeons with experience in laparoscopic surgery. This may be related to the use of the handles and other controls. It is worth noting that the Versius^TM^ robotic platform does not have pedals, so all controls are integrated into the handles (e.g., clutch), substantially increasing their use compared to other platforms such as da Vinci^TM^ (Intuitive Surgical; Sunnyvale, CA, USA) that do have pedals for camera control and active instruments. Another study in colorectal surgery demonstrated prolonged periods of low-intensity muscle activity in the shoulders during laparoscopic surgery and in the forearms for robotic-assisted surgery [25]. RULA analysis indicated a greater need for a change in working posture during laparoscopic compared to robotic surgery.

On the other hand, muscle workload analysis helps to optimize the surgeon’s posture and minimize fatigue. Prolonged surgical procedures, such as laparoscopic and robotic surgeries, can lead to significant muscle fatigue in surgeons [26]. This affects cognitive function, decision making, and overall performance during surgery. By understanding muscle fatigue, ergonomic improvements can be made to surgical techniques and equipment design, and strategies can be implemented to mitigate its impact on surgeon well-being and surgical outcomes [27]. The results of the present study explore a comparison between conventional and robotic-assisted laparoscopic surgeries from the perspective of localized muscle fatigue and muscle activity.

Joint EMG spectrum and amplitude analysis (JASA) graphs have been widely applied in the scientific literature for the analysis of muscle fatigue [28,29]. This method relates the electrical activity and median frequency of the EMG spectrum, obtaining in a schematic representation of the relationship between localized muscle fatigue and force use [30].

In previous studies, trapezius muscle fatigue during laparoscopic surgeries has been studied, as the trapezius is shown to be vulnerable to localized muscle fatigue, being the main muscle affected in the process of musculoskeletal tension [31,32]. This fact is being corrected with robotic surgery as the movement of the main dominant muscles for laparoscopic surgery has been seen to be reduced, supporting the advantages of robotic surgery [33]. In addition, these results show less localized muscle fatigue and greater use of force with robotic surgery than with conventional laparoscopic surgery. In the present study, the results showed great similarity to those obtained by novice surgeons. However, in the case of expert surgeons, force use and localized muscle fatigue were reduced for conventional laparoscopic surgery but high force use and slightly higher localized muscle fatigue were seen for robotic-assisted surgery. This fact could be related to the high degree of experience in performing conventional laparoscopic surgeries in contrast to robotic-assisted surgeries [34].

The results obtained during simulator tasks for expert and novice surgeons are in alignment with those obtained by Wee et al. [35], who demonstrated that from an ergonomic point of view robotic surgery is superior to conventional laparoscopic surgery. Therefore, future robotic surgeons should receive formal and thorough training for adequate familiarization [34].

Muscle activity is closely related to muscle fatigue, and prolonged muscle activity of small, single muscle fibers can cause degenerative muscle changes even with very low levels of muscle activity. For this reason, ergonomic positioning is very important for laparoscopic surgical activities [36]. The muscles analyzed in this study have been extensively evaluated in other works for laparoscopic and robotic surgeries, the main muscles being the upper and middle trapezius, triceps brachii, and brachioradialis [37]. Overall, for both types of surgeons, expert and novice, conventional laparoscopic surgery required greater muscle activity for the middle trapezius and right-sided triceps brachii. Robotic-assisted surgery required greater muscle activity for the vastus lateralis on the left side.

From the results of the present study, it stands out that muscle activity on the right side is lower than muscle activity on the left side. This fact could be related to the dominant side of the surgeons, showing less effort using the dominant side than the non-dominant side. Consistent with this fact, muscle activity on the dominant side also appears to increase slightly over time compared with the non-dominant side, suggesting that the dominant side may be more susceptible to slowly developing future musculoskeletal disorders [38].

Considering the surgeons’ experience, expert surgeons required greater muscle activity in robotic-assisted surgery than in conventional laparoscopic surgery. However, novice surgeons required greater muscle activity in conventional laparoscopic surgery than in robotic-assisted surgery. This could be related to the fact that expert surgeons have a high degree of experience in performing conventional laparoscopic surgeries in contrast to robotic-assisted surgeries [34] and yet novice surgeons have more developed skills necessary for robotic-assisted surgery and hand–eye coordination skills. Moreover, all muscles bilaterally show statistically significant differences between laparoscopic and robotic-assisted surgeries. This fact demonstrates the different use of muscles between laparoscopic and robotic-assisted surgeries for both types of surgeons, experts and novices.

Among the limitations of this study is the small number of participants. It is necessary to continue working in this line of research, increasing the number of studies to obtain more conclusive and representative results. On the other hand, due to the limitations due to the number of EMG sensors to be used simultaneously, there are still important muscle groups related to laparoscopic surgery, such as the deltoids or the biceps brachii, that should be evaluated in future studies to obtain more comprehensive results. Finally, it would be necessary to study the correlation over time between the activity of the muscle groups and the joint angles to analyze whether a posture maintained over time may be related to the appearance of localized muscle fatigue or another musculoskeletal disorder.

## 5. Conclusions

In this study, a set of wearable technologies and devices have been implemented and developed to record and analyze physiological and ergonomic parameters of surgeons during surgical performance in conventional and robotic-assisted laparoscopic surgery. Robotic-assisted laparoscopic surgery showed better performance and better ergonomic outcomes for surgeons than conventional laparoscopic surgery. Regarding physiological parameters, the objective stress experienced during conventional laparoscopic procedures was higher for both physiological recordings (EDA and ECG), related to the subjective questionnaire for stress (SURG-TLX), and for mental and physiological demand, allowing robotic-assisted laparoscopic surgery to present lower stress levels. In addition, surgeon motion analysis in robotic-assisted laparoscopic surgery contributes to better outcomes, reduced fatigue, and improved safety and health than conventional laparoscopic surgery, both for surgeons and patients. Regarding ergonomic parameters, postural habits and motor control are different among surgeons, which affects muscle activation patterns. Understanding muscle activity during robotic-assisted laparoscopic surgery ensures precise movements, minimizes fatigue, and contributes to improved patient outcomes and surgeon health.

Future studies focusing on the robotic work environment, both the lead surgeon and the rest of the surgical team, should be strengthened. It would be convenient to extend the studies on complex surgical procedures to obtain more conclusive results regarding the ergonomic and stress of surgeons in situations close to reality and for different surgical disciplines. Similarly, new cognitive analysis methods such as eye tracking or electroencephalography (EEG) should be studied and compared with the ergonomic and physiological results obtained by using the methodology proposed in this work. Finally, it would be interesting to compare different robotic platforms for laparoscopic surgery and with different configurations with respect to the controls, the type of vision system, or the use of foot pedals and how they affect the surgeon’s ergonomics during surgical performance.

Ergonomic analysis allows us to study individual variations in the different ergonomic parameters to improve surgical training and guidelines. Considering the rapid expansion of robotic-assisted surgical procedures in clinical practice, the analysis and improvement of the ergonomic conditions of surgeons and the surgical team are essential to provide optimal working conditions, ensuring the well-being of the surgical team and thus the quality of patient care. Training of the surgical team in optimizing ergonomic settings may be necessary to maximize ergonomic benefits in surgical robotics.

## Figures and Tables

**Table 1:** Comparison of RULA score during conventional laparoscopic (CONV) and robotic-assisted (ROB) suturing on simulator for novice and experienced laparoscopic surgeons.

	Experience	Technique	Value
Upper limbs	Novices	CONV	4.333 ± 0.577
		ROB	3.500 ± 0.707
	Experienced	CONV	4.000 ± 1.414
		ROB	3.000 ± 0.000
Body and lower limbs	Novices	CONV	7.000 ± 0.000
		ROB	5.500 ± 2.121
	Experienced	CONV	6.000 ± 1.414
		ROB	5.000 ± 1.414
Global score	Novices	CONV	6.333 ± 0.577
		ROB	4.000 ± 1.414
	Experienced	CONV	5.500 ± 2.121
		ROB	5.000 ± 2.828

**Table 2:** Comparison of RULA score during different surgical procedures performed by conventional laparoscopy (CONV) and robotic-assisted surgery (ROB).

	Procedure	Technique	Value
Upper limbs	Gastrotomy	CONV	3
		ROB	3
	Total nephrectomy	CONV	3
		ROB	3
	Ovariectomy	CONV	3
		ROB	4
Body and low limbs	Gastrotomy	CONV	7
		ROB	7
	Total nephrectomy	CONV	3
		ROB	3
	Ovariectomy	CONV	7
		ROB	7
Global score	Gastrotomy	CONV	6
		ROB	6
	Total nephrectomy	CONV	4
		ROB	4
	Ovariectomy	CONV	6
		ROB	7

## Data Availability

Data available on request due to restrictions e.g. privacy or ethical.
